# Epithelial-to-Mesenchymal Transition of RPE Cells *In Vitro* Confers Increased β1,6-*N*-Glycosylation and Increased Susceptibility to Galectin-3 Binding

**DOI:** 10.1371/journal.pone.0146887

**Published:** 2016-01-13

**Authors:** Claudia S. Priglinger, Jara Obermann, Christoph M. Szober, Juliane Merl-Pham, Uli Ohmayer, Jennifer Behler, Fabian Gruhn, Thomas C. Kreutzer, Christian Wertheimer, Arie Geerlof, Siegfried G. Priglinger, Stefanie M. Hauck

**Affiliations:** 1 Department of Ophthalmology, Ludwig-Maximilians-University, Munich, Germany; 2 Research Unit Protein Science, Helmholtz Zentrum Munich, German Research Center for Environmental Health (GmbH), Munich, Germany; 3 Protein Expression and Purification Facility, Institute of Structural Biology, Helmholtz Zentrum Munich, German Research Center for Environmental Health (GmbH), Neuherberg, Germany; Universidade de São Paulo, BRAZIL

## Abstract

Epithelial-to-mesenchymal transition (EMT) of retinal pigment epithelial cells is a crucial event in the onset of proliferative vitreoretinopathy (PVR), the most common reason for treatment failure in retinal detachment surgery. We studied alterations in the cell surface glycan expression profile upon EMT of RPE cells and focused on its relevance for the interaction with galectin-3 (Gal-3), a carbohydrate binding protein, which can inhibit attachment and spreading of human RPE cells in a dose- and carbohydrate-dependent manner, and thus bares the potential to counteract PVR-associated cellular events. Lectin blot analysis revealed that EMT of RPE cells in vitro confers a glycomic shift towards an abundance of Thomsen-Friedenreich antigen, poly-N-acetyllactosamine chains, and complex-type branched N-glycans. Using inhibitors of glycosylation we found that both, binding of Gal-3 to the RPE cell surface and Gal-3-mediated inhibition of RPE attachment and spreading, strongly depend on the interaction of Gal-3 with tri- or tetra-antennary complex type N-glycans and sialylation of glycans but not on complex-type O-glycans. Importantly, we found that β1,6 *N*-acetylglucosaminyltransferase V (Mgat5), the key enzyme catalyzing the synthesis of tetra- or tri-antennary complex type N-glycans, is increased upon EMT of RPE cells. Silencing of Mgat5 by siRNA and CRISPR-Cas9 genome editing resulted in reduced Gal-3 binding. We conclude from these data that binding of recombinant Gal-3 to the RPE cell surface and inhibitory effects on RPE attachment and spreading largely dependent on interaction with Mgat5 modified N-glycans, which are more abundant on dedifferentiated than on the healthy, native RPE cells. Based on these findings we hypothesize that EMT of RPE cells *in vitro* confers glycomic changes, which account for high affinity binding of recombinant Gal-3, particularly to the cell surface of myofibroblastic RPE. From a future perspective recombinant Gal-3 may disclose a therapeutic option allowing for selectively targeting RPE cells with pathogenic relevance for development of PVR.

## Introduction

Proliferative vitreoretinopathy (PVR) is the major cause of treatment failure in retinal detachment surgery even after primarily successful re-attachment of the retina. In PVR scar-like fibrocellular membranes are formed on the surfaces of the neuroretina, which contract and lead to tractional retinal (re-)detachment and ultimately permanent loss of vision. In the past, pharmacologic prevention of PVR membrane formation has primarily been based on antiproliferative strategies or the inhibition of growth factors and inflammation [1–4]. However, the results from most approaches have been disappointing, largely because of their ineffectiveness or toxicity to the healthy tissue [5–8]. Studies targeting growth factors or inflammatory cytokines showed that PVR is a primarily cell driven disease that cannot be controlled by the inhibition of single growth factors alone. Thus, a need remains for nontoxic agents that will specifically block cellular activities such as RPE attachment or spreading in PVR and, even more importantly, selectively target the cells of relevance for the pathogenesis of the disease. One promising approach involves the use of endogenous effectors that normally exert control over cell movement or proliferation. In the present study we focus on such a concept.

Epithelial-to-mesenchymal transition (EMT) of retinal pigment epithelial (RPE) cells to a myofibroblastic phenotype followed by attachment, spreading, migration and proliferation on the retinal surfaces is the key cellular event in the onset of PVR [9,10]. Only in recent years it has been recognised that upon EMT alterations in cell surface glycosylation occur in malignant and premalignant epithelia [11] and that these glycomic changes can influence the signals elicited by endogenous carbohydrate binding proteins such as galectins, selectins or siglecs. In the current understanding the different subtypes of lectins by virtue of their oligosaccharide specificity decipher the information stored in the glycan chains on the cell surface. This in turn is dependent on the respective glycomic state of the ligands. Thus, because glycan structures present on the counterreceptors may change with the biological context, lectins can negatively or positively regulate cellular processes [12].

Galectins are animal lectins that exhibit selective affinity for β-galactoside containing moieties [13]. Galectin-3 (Gal-3), a ubiquitously expressed member of the galectin family, is overexpressed in many malignant and premalignant tissues [14], in migrating corneal epithelial cells [15] and in transdifferentiated RPE cells [16]. Gal-3 can be found in the cytosol and nucleus, and is secreted by a nonclassical pathway to the cell surface [17]. Cytoplasmic Gal-3 is known to act anti-apoptotic [18,19] and positively influences cell growth [20]. Nuclear Gal-3 contributes to pre-mRNA splicing [21]. Extracellular Gal-3 is involved in cell-cell and cell-matrix interactions [12,22], such as adhesion, migration or spreading of cells.

Extracellular Gal-3 interacts with glycoconjugates on glycoproteins present on the cell surfaces or in the ECM. In general, galectins bind *N*-glycans in proportion to the amount of N-acetyllactosamine (LacNAc) in the glycan, which is determined by the number LacNAc branches (reflecting the action of the glycosyltransferases N-acetylglucosaminyltransferase I, II, IV and V, encoded by *Mgat1*, *Mgat2*, *Mgat4a/b*, *and Mgat5*) as well as poly-LacNAc extension [23]. Among the branched *N*-glycans, the β1,6GlcNAc-branched product of Mgat5 is the preferred acceptor for further extension with N-actyllactosamine units, which increase the affinity for Gal-3. However, Gal-3 is known to bind also to O-glycan structures such as the unsubstituted Thomsen-Friedenreich (TF) antigen (Galβ1,3GalNAc, i.e. the core-I disaccharide of O-glycans) [24], and Gal-3 binding can further be modified by the presence of sialic acid residues [25]. By binding to the glycoconjugates, galectins can deliver signals intracellularly as well as mediate cell-cell and cell-ECM adhesion [26].

In previous studies we observed an inhibition of RPE attachment and spreading by Gal-1 and Gal-3 and found that recombinant Gal-1- and Gal-3 bind to the RPE cell surface in a dose-dependent and carbohydrate-dependent manner [16,27]. Acting as a cross-linking lectin on the surface of mobilized RPE, Gal-3 significantly disturbed microfilament assembly and, in correlation with decreased attachment, inhibited ERK phosphorylation [16]. We identified CD147 and α3β1-integrin as RPE-specific glycoprotein counterreceptors of Gal-3 [28], however, the nature of the corresponding saccharide ligands remained elusive.

In the present study we investigated the glycomic changes associated with EMT of RPE cells in vitro, defined the saccharide ligands on the surface of transdifferentiated RPE cells, which account for Gal-3 mediated inhibition of RPE attachment and spreading, and report expression changes of the respective glycosyltransferase. We report here for the first time that myofibroblastic RPE cells compared with native, epitheloid RPE cells confer an upregulation of distinct glycans that may account for high affinity binding of Gal-3 to myofibroblastic but not to healthy, native RPE cells. From a therapeutic perspective these data further corroborate the suitability of recombinant galectins or galectin-mimetics with similar carbohydrate-binding-specificity for prophylaxis of PVR. Further investigation of this pathway may aid in development of a galectin-based approach for prophylaxis of PVR or galectin-based targeted drug delivery.

## Materials and Methods

### Cell lines

Human retinal pigment epithelial (RPE) cells were isolated from human cadaver eyes obtained from the Eye Bank of the Department of Ophthalmology at the Linz General Hospital (Linz, Austria) or at the LMU Munich (Munich, Germany) and processed within 8 to 24 h after death. Donor age ranged from 21 to 80 years and none of the donors had a known history of eye disease. Methods for securing human tissue were humane, included written informed consent and approval of the relatives, and complied with the Declaration of Helsinki. Informed consents were kept in a repository at the respective eye banks. The isolation of RPE cells from human cadaver eyes for this study and the respective consent procedure were approved by the ethics committee of the Klinikum of the Ludwig-Maximilians-University Munich and the ethics committee of the Land Oberoesterreich. In brief, human postmortem donor eyes were enucleated by an ophthalmologist according to the institutions standard operating procedures. After removal of the corneoscleral ring for cornea-transplantation the posterior pole of the eye was processed for isolation of primary human RPE cells. Human RPE cells were harvested from sixteen post-mortem eyes following the procedure as described previously [29,30]. Primary RPE cells were subcultured and maintained in Dulbeccos modified Eagles medium (DMEM; Biochrom, Berlin, Germany) supplemented with 10% fetal calf serum (FCS; Biochrom) at 37°C and 5% CO_2._ Primary human RPE cells of passage 3 to 7 were used for experiments. When indicated, adult porcine eyes were provided by a local slaughterhouse, with procedures supervised and approved by the local veterinarian. They were removed from the animals within 5 min after death and kept on ice in CO_2_-independent medium (Invitrogen) until further use. RPE cells were then isolated using the same procedure as described above for human RPE cells. Native porcine RPE cells were either processed for flow cytometry immediately or placed into culture until cells have undergone EMT. RPE cells from eyes of twenty different pigs were used in this study, thirteen replicates of native cells and three biological replicates of passage-3 cells. For transfection experiments the immortalized human RPE cell line ARPE-19 (ATCC, No. CRL-2302™) and the highly transfectable derivative HEK293T embryonic kidney cell line (ATCC, HEK293T, CRL-3216™) were used and maintained in DMEM/Ham´s F12 with 10% FCS and DMEM (Life Technologies) supplemented with 2 mM L-glutamine and 10% FCS, respectively.

### Human Galectin-3: expression, purification, labeling and quality controls

Human Gal-3 was cloned in the bacterial pETM-11 expression vector as previously described [28]. pETM-11/hgalectin3 was transformed into the *E*. *coli* strain BL21 (DE3) and cultured at 20°C in 2-L flasks containing 500 ml ZYM 5052 auto-induction medium [31] and 100 μg/ml kanamycin. Cells were harvested by centrifugation after reaching saturation, divided in 3 equal pellets and stored at -20°C. One pellet of each construct was resuspended in 30 ml lysis buffer (20mM Tris-HCl, 150mM NaCl, 10mM MgSO_4_, 10 μg/ml DNaseI, 1mM AEBSF.HCl, 0.03% (v/v) CHAPS, 1 mg/ml lysosyme, pH 7.5), and lysed by sonication. The lysates were clarified by centrifugation (40,000 x g) and filtration (0.2 μm). The supernatants were applied to 2-ml lactose-agarose columns (J-Oil Mills, Tokyo, Japan), equilibrated in buffer A (20mM Tris-HCl, 150mM NaCl, 0.03% (v/v) CHAPS, pH 7.5). The columns were washed three times with 25 ml buffer A and bound proteins were eluted two times with 5 ml buffer A containing 0.2 M lactose. This procedure was repeated twice and protein containing fractions were pooled and dialyzed overnight at 4°C against 1 L PBS. The dialysates were filtered (0.2 μm) and stored at 4°C. Protein concentration was determined by measuring the absorbance at 280 nm using specific absorbances for His_6_-tagged hGalectin-3 of 1.332 nm. For biotinylation, 2 mg of purified Gal-3 were dialyzed overnight at 4°C against 0.1M sodiumhydrogencarbonate, pH 8.0, followed by a 1 hour dialysis against the same buffer, but with pH 9.2. Gal-3 was then biotinylated for 1 hour at RT with 200 μg biotinamidohexanoic acid N-hydroxysuccinimide ester according to the manufacturer´s instructions (Sigma, Taufkirchen, Germany) followed by dialysis against PBS overnight. Biotinylation was confirmed in western blot analysis by detection with HRP-coupled streptavidin (Roche, Mannheim, Germany).

### Lectin blot analysis

Native and cultured human RPE cells of passage 3 were lysed in RIPA cell lysis buffer as described above. Equal total protein amounts (7.5 μg) were electrophoresed on 10% polyacrylamide gels and transferred by semidry blotting to polyvinyl-difluoride membranes (GE-Healthcare, Freiburg, Germany). Unspecific binding was blocked with 1x carbohydrate-free blocking solution (Vector Labs) for one hour at RT. Blots were probed with biotin-conjugated Phaseolus vulgaris leukoagglutinin (PHA-L), or Phaseolus vulgaris erythroagglutinin (PHA-E), or Concavalin A (ConA), Wheat germ agglutinin (WGA), or Ricinus communis agglutinin (RCA), or Sambuccus nigra lectin (SNA), or Griffonia simplificolia lectin-1 (GSL-1), or Peanut agglutinin (PNA), or Maackia amurensis lectin (MAL-2), or Lycopersicum esculentum lectin (LEL), or Jacalin (JAC), respectively (all from Vector Labs), diluted 1:1,000 in PBS 0.05% Tween 20 overnight at 4°C. Membranes were then washed three times in PBS and the lectin-reactive components were visualized using streptavidin-POD (1:15,000) for 1h at RT. Staining for GAPDH content with monoclonal mouse anti-GAPDH antibody (Millipore (MAB374), dilution 1:10,000) and the respective secondary antibody (peroxidase-coupled goat anti-mouse IgG, Jackson, dilution 1:75,000) served as loading control. Protein signals were then visualized using ECL Prime WB Detection Reagent plus enhanced chemiluminescence kit (GE Healthcare) and signals were captured on Hyperfilm ECL (GE Healthcare). All experiments have been repeated at least three times.

### Flow cytometry

Subconfluent monolayers of cultured, myofibroblastic human RPE cells were treated with 1 μg/mL Swainsonine (Sigma) in methanol, or 1 mM deoxymannojirimycin (DMNJ, Calbiochem) in a.d., or 2 mM benzyl-α-*N*-acetylgalactosamine (Benzyl-GalNAc) in ethanol (Calbiochem) or the respective buffer controls for 72 hours, or 0.2 mU/mL Vibrio Cholera neuraminidase (Roche) in DMEM for 30 minutes at 37°C and 5% CO_2_. Cells were resuspended in trypsin/EDTA and cell staining was performed in 96 well round-bottom plates with 2×10^5^ cells per well. Cells were either incubated with biotinylated recombinant Gal-3 (30 μg/mL) or biotin-conjugated PHA-L, or PHA-E, or PNA, or WGA, or ConA, or MAL-2, or SNA for 30 minutes on ice. Recombinant human Gal-5 (R&D systems) and SJA, which do not bind to human RPE cells, or cells incubated with avidin-FITC alone served as negative controls. Native and cultured porcine RPE cells were processed for FACS analysis as described for human RPE cells. All plant lectins were biotin-conjugated and obtained from Vector laboratories and used at a concentration 3 μg/ml. After another three washes in PBS, surface bound biotinylated lectins were stained with streptavidin-FITC (dilution 1:200; Southern Biotech, Birmingham, Alabama, USA) in staining buffer for 30 min at 4°C. Cells were kept at 4°C in staining buffer with 1% PFA until processing. 10,000 cells were measured per staining. Data were acquired on FACS Canto II with FACS Diva 6.1.3 software (both BD Biosciences, Heidelberg, Germany).

### Cell adhesion assay

Surfaces of 96-well plates (Nunc, Wiesbaden, Germany) were coated with fibronectin (Fn) solubilized in 1× PBS (pH 7.4) to yield a final density of 5 μg/cm^2^. The plates were air dried, washed with 1× PBS and preincubated with 1% BSA/PBS for one hour before plating the cells. Prior to the assay, when indicated, subconfluent, myofibroblastic RPE cells were pretreated with 2 mM benzyl-α-N-acetylgalactosamine (benzyl-GalNAc) in DMSO or 150 μM deoxymannojirimycin (DMNJ) dissolved in PBS (both from Calbiochem) or with the appropriate buffer controls for 72 hours at 37°C and 5% CO_2_. Trypsinized RPE cells were then washed once in DMEM with 10% FCS to quench trypsin activity. Suspensions of 2×10^4^ cells were incubated in the presence or absence of 125 μg/mL Gal-3 for 30 min at 37°C and 5% CO_2_. Cells were then added to the wells and allowed to attach for 1 h at 37°C and 5% CO_2_. The extent of adherence was measured by MTT assay as previously described [16,32]. The number of attached living cells was proportional to the absorbance of Formazan at 550 nm, as determined by a scanning multiwell spectrophotometer (Molecular Devices). Experiments were performed in triplicate and repeated at least three times.

### Cell spreading assay

Cell spreading was assayed on four chamber slides coated with Fn to yield a final concentration of 5 μg/cm^2^. Before the experiment, myofibroblastic RPE cells were cultured in the presence or absence of DMNJ or benzyl-GalNAc as described for the cell adhesion assay. Cells were dettached by trypsinization, which involved 0.25% trypsin in 5 mM EDTA, washed once in DMEM with 10% FCS, once with medium containing 0.4% FCS, and then incubated with or without 125 μg/mL Gal-3 for 30 minutes at 37°C. Thereafter cells were resuspended and plated at a concentration of 2.5×10^4^ cells per well and allowed to spread for 3.5 h at 37°C and 5% CO_2_ [16,32]. Cells were washed three times in PBS, fixed with methanol, stained with Giemsa, and mounted in Kaiser gelatin (Merck). To quantify cell spreading, four separate fields were photographed using phase contrast microscopy. Cells were visually inspected (by a blinded observer) and graded into either spread cells displaying a clearly defined halo of cytoplasm around the nucleus and non-spread cells with no clear cytoplasmic area. Percentage of spread and non-spread cells were calculated. Each experiment was performed in duplicate wells and was repeated at least three times.

### Silencing of Mgat5 expression by siRNA

For posttranscriptional gene-silencing experiments a set of three different siRNAs targeting human MGAT5 (ID 4249, OriGene, USA) was tested for silencing of Mgat5 expression. For this purpose, we used ARPE19 cells, which allow for excluding inter-experimental variables, which may occur when employing primary RPE cell lines derived from different donors and different passages. RPE cells were trypsinized the day before transfection, diluted with fresh medium without antibiotics, and plated on 35-mm dishes or four chamber slides to reach 50% to 60% confluence the next day. Transient transfection with siRNAs was performed using Torpedo^siRNA^ transfection reagent™ (Ibidi, Martinsried, Germany) according to the manufacturer´s protocol. In brief, for transfection of cells grown in 35 mm dishes 7.0 μL of Torpedo^siRNA^ transfection reagent™ were preincubated in 120 μL 1x transfection buffer together with 2.5 or 5.0 μL of siRNA stock solution, giving a final siRNA concentration of 50 pmol or 100 pmol, respectively. The mixture was allowed to sit for 15 min at RT for complex formation. The reagent complex was then gently added to the cells in a total volume of 1 mL DMEM/10% FCS. Control cells were either transfected with a nonspecific scrambled siRNA duplex or with transfection reagent and buffer instead of siRNA. After 48 hours cells were harvested and processed for immunoblotting. Gal-3 binding experiments were performed 96 hours after transfection. Specific silencing was confirmed by at least three independent experiments.

### Silencing of Mgat5 expression by Lenti-CRISPR/Cas9

The knockdown of Mgat5 in ARPE19 cells was performed by CRISPR/Cas9 (clustered regularly interspaced short palindrome repeats) guided genome editing. Lentiviral particles were used as vectors to integrate an expression cassette into the genome of the ARPE19 cells. This cassette codes for the guide RNA (gRNA—guides the nuclease to the respective gene), the nuclease Cas9, and a marker protein (puromycin resistance). The whole workflow is based on publications from the Zhang group [33–35]. To select an appropriate gRNA the CRISPR design tool was used (http://crispr.mit.edu/) [35]. The sequence of the gRNAs (20 nucleotides in length) for Mgat5 was 5´-GTGGTGGATGGGCCATACGC-´3. Lentiviruses were created in HEK293T cells (Life Technologies) by co-transfection of three plasmids as described in [33,34]. Briefly, for each gRNA expression vector, two oligonucleotides were created (as described in Sanjana et al. 2014 and protocols published at http://genome-engineering.org/gecko/), phosphorylated, annealed, and ligated with the BsmBI (Thermo Fisher) digested vector “lentiCRISPRv2” (addgene #52961) [33]. For the production of each lentivirus, 80% confluent HEK293T cells were co-transfected in 8 ml OptiMEM (Life Technologies) with 5μg and 7.5μg of packaging plasmids pMD2.G (addgene #12259) and psPAX2 (addgene #12260), respectively, and 10μg of the transfer plasmid (see above), using 100 μl Plus Reagent™ (Life Technologies) and 50μl Lipofectamine 2000™ (Life Technologies). Medium was changed after 6 hours to DMEM (Life Technologies) with 10% fetal bovine serum (Life Technologies) and 1% bovine serum albumin (GE Healthcare). After 60 hours, lentivirus containing supernatants were harvested by centrifugation at 4°C and 2000g for 15 minutes and purified by 0.45μm filtering. ARPE-19 cells grown in 10 cm^2^ dishes were transduced directly with 250 μl of lentivirus containing supernatant. After one day, 2 μg/ml puromycin was added to the media to select transduced cells. As control, a lentivirus produced from plasmid “lentiCRISPRv2” (addgene #52961) was created, which codes for the nuclease Cas9 and the selection marker but does not code for a functional gRNA but a 2kb “filler” sequence, which is not incorporated into a functional ribonucleoprotein complex of RNA and Cas9. Mgat5 knockout and reduced Gal-3 binding to the transfected cells was assessed by western blot analysis and FACS analysis 16 days after transfection in order to allow for validation of sufficient knock-down of the target gene expression.

### Western blot analysis

For preparation of protein lysates of native RPE cells the posterior poles from eyes of twenty different human donors were prepared as described above. RPE cells were released from Bruch’s membrane by gently pipetting ice cold phosphate buffered saline (PBS, pH 7.4) solution into the eye cup. The suspended RPE cells were transferred to a 35 mm^2^ petri dish and checked for cross contamination using a microscope. Cell suspensions were then transferred to a 2.0 ml microcentrifuge tube and centrifuged for 5 min at 800 rpm. After centrifugation the supernatant was removed and replaced by RIPA cell lysis buffer (50 mM Tris, pH 8.0; 150 mM NaCl; 1% NP40; 0.5% deoxycholate; 0.1% SDS) containing an appropriate amount of protease inhibitors (Complete Mini, Roche, Mannheim, Germany). After centrifugation for 30 minutes at 12.000 rpm in the cold, the supernatant was transferred to fresh tubes and stored at −20°C for future use.

Cultured human RPE cells were washed twice with ice-cold PBS, and lysed in RIPA as described above. The protein content was measured using bicinchoninic acid (BCA) protein assay reagent (Pierce, Rockford, IL). Equal total protein amounts (50 μg) were electrophoresed on 10% polyacrylamide gels and transferred by semidry blotting to polyvinyl-difluoride membranes (GE-Healthcare). Unspecific binding was blocked with 5% non-fat dry milk in PBS containing 0.1% Tween 20 (PBST; pH 7.2) for one hour at RT. Blots were probed with mouse anti-Mgat5 (MAB5469, R&D Systems) at a final dilution of 1:250, or rat anti Tyr-tubulin (ab6160, Abcam) at a final dilution of 1:10,000, or mouse anti-GAPDH antibody (MAB374, Millipore) at a final dilution of 1:10,000 and allowed to react overnight at 4°C.

Appropriate secondary horseradish peroxidase-coupled antibodies (JacksonImmunoResearch, Newmarket, UK) were used in a dilution of 1:15,000. Protein signals were then visualized using ECL Prime WB Detection Reagent (GE Healthcare) and signals were monitored on the chemiluminescence imaging system Fusion FX7 (Vilber Lourmat Gmbh) and analyzed with the corresponding software Fusion 15.18 (Vilber Lourmat). All experiments have been repeated at least three times.

### RNA isolation and real-time quantitative RT-PCR

Total RNA was isolated from 35 mm petri dishes by the guanidium thiocyanate-phenol-chloroform extraction method (Stratagene, Heidelberg, Germany) as described [29]. cDNA was synthesized from total cellular RNA using the First Strand Synthesis Superscript III Reaction mix™ from Invitrogen-Life Technologies according to the manufacturer´s protocol. In brief, one μg of total RNA was reverse transcribed in a 20 μL volume containing 10 μL 2x RT Reaction Mix™ and 2 μl Enzyme Mix™ for 1 hour at 42°C. cDNA was then incubated at 85°C for 5 minutes followed by addition of 1 μL RNase inhibitor (all from Invitrogen-Life Technologies) for another 20 minutes at 37°C. Quantitative real-time PCR was performed on a sequence-detection system (LightCycler 1.5™, Roche) using heat-activated *Taq* DNA polymerase (LightCycler TaqMan Master™; Roche), according to the manufacturer´s protocol. Primers and respective probes were designed with the probe finder software on the Roche Applied Biosciences website (https://qpcr1.probefinder.com/roche2.html). The primer pairs used were: Mgat5 (left ´5-GCTCATCTGCGAGCCTTCT-3´, positions 2004–2022; right ´5-TCTGAGCTTTGGCAGGTCA-3´; positions 2069–2087), ß2-microglobulin (left ´5-TTCTGGCCTGGAGGCTATC-3´, positions 58–76; right ´5-TCAGGAAATTTGACTTTCCATTC-3´, positions 121–143). The cycling conditions were as follows: after an initial hold of 2 minutes at 50°C and 10 minutes at 95°C, the samples were cycled 40 times at 95°C for 15 seconds and 60°C for 60 seconds. The quantity of mRNA expression was analysed by standard curve quantification for the target gene and the ß2-microglobulin mRNA in the same sample using the Light Cycler Relative Quantification Software™ package (Version 1.0; Roche). All measurements were performed in duplicate. Controls consisting of bi-distilled H_2_O were negative in all runs. Experiments were repeated at least three times.

### Immunocytochemical Gal-3 localization

RPE cells were grown in four chamber slides and were maintained in DMEM supplemented with 10% FCS until 96 hours after transfection. Cells were washed three times (three minutes each wash) in 0.1% BSA (BSA) and phosphate buffered saline (PBS; 137 mM NaCl; 2.7 mM KCl, 10 mM Na_2_HPO_4_; 2 mM KH_2_PO_4_; pH 7.4) at room temperature. Thereafter, cells were incubated with biotinylated Gal-3 (60 μg/mL) in DMEM for 30 minutes at 37°C. After three washes in PBS (5 minutes each wash), cells were fixed in 4% paraformaldehyde for 5 minutes on ice. After another three washes in PBS, cells were incubated in Streptavidin Alexa-Fluor^488^ conjugate™ (Molecular probes/Invitrogen, Paisley, UK) diluted 1:1000 in PBS for 1 hour at RT in the dark. After three washes in PBS cells were mounted in fluorescent mounting medium with DAPI four counterstaining of nuclei (DAKO, Hamburg, Germany). Cells incubated with Streptavidin Alexa-Fluor^488^ conjugate™ alone served as negative controls. Gal-3 dependent signals were monitored on a fluorescence microscope (Leica, Wetzlar, Germany).

### Lectin histochemistry of intact eyecups

For lectin histochemistry the retina was carefully peeled away and the eye cups were then filled with PBS/EDTA for 15 minutes at room temperature. After incubating the posterior poles in 0.1% PBS/BSA for 5 minutes, one eye cup of each donor pair was filled with 60 μg/mL recombinant biotinylated Gal-3 in DMEM and incubated for 30 minutes at 37°C, whereas the fellow eye was incubated under the same conditions without Gal-3 in the medium. The eye cups were then rinsed three times with PBS to remove unbound Gal-3 (5 minutes each wash), transferred to 4% formaldehyde and processed for embedding in paraffin. Paraffin-embedded RPE/choroid/sclera specimens were cut into sections of 8 μm each, collected on glass slides and incubated at 42°C for 48h to prevent washing off the sections during the process of histochemical staining. In order to remove the paraffin and to rehydrate the sections, they were first rinsed with xylene (once for 5 min) and isopropanol (twice for 5 min), followed by ethanol (96% and 70%—each for 5 min) and finally millipore water (5 min). Prior to incubation with plant lectins, sections were blocked for 45 minutes in 1x carbohydrate-free blocking solution (Vector laboratories) and streptavidin blocking solution (Streptavidin/Biotin Blocking Kit, Vector laboratories) followed by addition of an appropriate amount of biotin solution for 15 minutes, in order to prevent non-specific staining. Sections were then incubated overnight at 4°C with 2 μg/mL biotin-conjugated PHA-L or biotin-conjugated ConA or streptavidin-peroxidase without prior addition of a plant lectin in order to visualize binding of biotinylated Gal-3 to native RPE cells in situ. After three washes in TBS-T (7 minutes each wash) streptavidin-POD (1:15,000, Sigma) was added for 15 minutes. Sections were then washed once in TBS and once in PBS (5 minutes each wash) before the Vector VIP Substrate-Kit™ reagent (Vector Laboratories) was added according to the manufacturer´s protocol. Sections were counterstained with Mayer´s Haemalaun and mounted with glass coverslips using Kaiser´s gelatine (Merck), and images were recorded with an Axio Imager from Zeiss and the Axio Vision 4.6 Vision software (both from Zeiss, Göttingen, Germany) at a total magnification of 400 fold.

### Statistical analysis

Statistical analysis was performed using XLSTAT 2015 software (MS Excel). The Mann–Whitney U test with a confidence interval of 95% was used to calculate statistically significant differences between samples. A p≤0.05 was considered statistically significant.

## Results

### Comparative glycomic profiling of native versus myofibroblastic RPE cells by plant lectins

**Fig 1:**
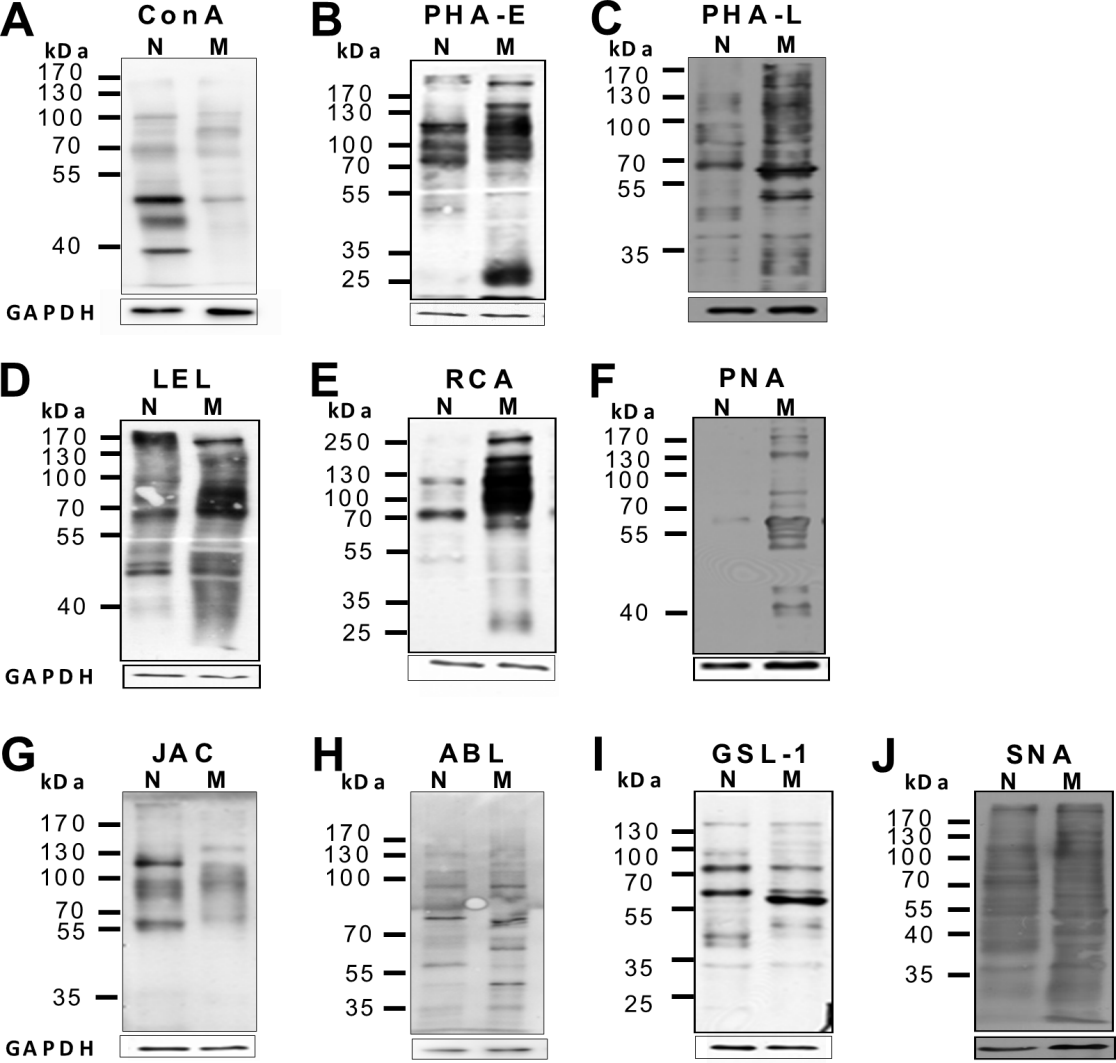
Glycomic profiling of native versus cultured, myofibroblastic RPE by lectin blot analysis reveals alterations in the glycan expression pattern upon EMT in vitro. Equal amounts of whole cellular protein lysates (7.5 μg) derived from native (N) and cultured, myofibroblastic (M) human RPE cells of passage 3 were separated by SDS page. Blots were incubated with the biotin-coupled plant lectins as indicated followed by peroxidase-coupled streptavidin (*large boxes*, *above*). Normalization to GAPDH (*small boxes*, *below*) shows equal total protein amounts of native RPE cells (N) and passaged RPE cells (M) were loaded in both slots. Molecular weight in kDa is indicated on the left (MW). The experiment was repeated at least three times with protein lysates from different donors of different age groups and different primary RPE cell lines of passage 3–7. A representative blot is shown.

Epithelial-to-mesenchymal transition (EMT) of RPE cells from a highly differentiated, epithelial cells to a myofibroblast-like phenotype is a hallmark event during pathogenesis of PVR and glycomic changes and altered glycogene expression have been reported to occur during EMT of other cell types [36,37]. To monitor changes of the glycan expression pattern during EMT of RPE cells, we profiled native, epithelial RPE and in vitro transdifferentiated, myofibroblastic RPE (having undergone EMT) for differential plant lectin binding. When RPE cells are cultured on plastic they undergo an EMT and fail to maintain a differentiated morphology. For this reason, cultured RPE cells are a well-accepted *in vitro* model for the fibroblast-like, wound-healing phenotype of RPE cells as found in early PVR [38] and were used in the present study. To monitor the overall complexity of RPE glycosylation, we first analyzed the presence of N-glycans using *Concavalin A* (ConA). ConA is a plant lectin, which binds avidly to the trimannoside core of N-glycans, bisected hybrid-type N-glycans and to a lesser extent to complex-type biantennary N-glycans, whereas highly branched N-glycans will not bind or bind not as tightly [15]. ConA recognized three pronounced bands in protein extracts from native RPE cells, whereas only faint bands were visible in cultured, myofibroblastic RPE (Fig 1A). Next we monitored for bisecting *N*-acetylglucosamine and the presence of β1,6-branching. *Phaseolus vulgaris erythroagglutinin* (PHA-E) specifically binds complex-type biantennary N-glycans and recognized several bands in native and dedifferentiated RPE cells with the most prominent bands in the 70 to 130 kDa region and an additional pronounced band at 25 kDa in only in cultured, myofibroblastic RPE (Fig 1B). In contrast, Phaseolus vulgaris leukoagglutinin (PHA-L), which has a high specificity for complex-type β1,6-branched tri- and tetrantennary *N*-glycans only weakly bound to extracts form native RPE cells, whereas strong signals were visible in the cell lysates derived from myofibroblastic RPE cells, consistent with an increase in β1,6-N-glycosylation in myofibroblastic, cultured RPE (Fig 1C). Overall, native RPE cells expressed complex-type biantennary N-glycans in a binding pattern comparably to that of myofibroblastic RPE with a tendency for a higher abundance PHA-E reactive glycans in myofibroblastic RPE. A clear difference concerned the relative abundance of ConA-reactive N-glycans in native RPE versus an increase in PHA-L-binding complex-type β1,6-branched tri- and tetrantennary *N*-glycans in myofibroblastic RPE.

Refining poly-N-acetyllactosamine chain extensions, LEL (*lycopersum esculentum lectin*), which binds internal *N*-acetyllactosamine within poly-N-acetyllactosamine chains [25], recognized several bands in native as well as dedifferentiated RPE cell extracts, although with a different pattern (Fig 1D). Whereas binding to native RPE was rather faint and diffuse, in extracts from myofibroblastaic RPE a polydisperse band between 100 and 130 kDa in blots from transdifferentiated, myofibroblastic RPE cells exhibited stronger reactivity. This may be indicative of pronounced chain extensions on a subset of glycoproteins in dedifferentiated RPE, although the possibility that these bands arise from the dual activity of LEL to high-mannose type N-glycans cannot be ruled out.

Next we determined the presence of terminal galactose residues and poly-N-acetyllactosamine chains. *Ricinus communis lectin* (RCA), a plant lectin that recognizes N- and O-glycans terminating in N-acetyllactosamine residues [25] strongly bound to extracts derived from transdifferentiated RPE cells with many bands between 70 and 180 kDa (Fig 1E) and an additional 250 kDa band. In general, the RCA binding patterns of myofibroblastic RPE cells strongly resembled that for PHA-E, although it was not identical. This was in clear contrast to the pattern found in native RPE cells. In native cells the PHA-E probe bound to three pronounced bands at approximately 80, 110 and 130 kDa, whereas RCA stained only a single band at about 80 kDa and one faint band at 130 kDa. These findings suggest that upon EMT of RPE cells an increase in chain end N-acetyllactosamine-units may particularly occur on complex-type (biantennary) N-glycans.

With respect to O-glycans we initiated our studies looking at the Thomsen-Friedenreich (TF)-antigen (galβ1,3 N-acetylgalactosamine alpha- (TF))^2^. The TF-antigen is the core I structure of mucin-type O-glycans and is recognized, although with limited specificity [39], by the plant lectin peanut agglutinin (PNA, *Arachis hypogea*). Only transdifferentiated RPE cells showed several PNA reactive bands, with one dominant band at approximately 60 kDa, which was hardly detectable in native RPE cells (Fig 1F). In contrast, jacalin (*Artocarpus integrifolia lectin;* Fig 1G) as well as agaricus bisporicus lectin (ABL; Fig 1H) supposed to bind sialylated as well as non-sialylated TF-antigen showed several bands in both, native and cultured RPE, with jacalin reacting with two major bands in native RPE, exclusively (Fig 1G). These findings may suggest that dedifferentiated RPE cells in contrast to native RPE cells express predominantly non-sialylated TF-antigen, whereas the sialylated form is found in both phenotypes. Using the N-acetylgalactosamine-binding lectin from *Griffonia simplicifolia* (GSL) two corresponding bands were found to carry the Tn (N-acetylgalactosamine-α-) antigen, with one additional pronounced band appearing in dedifferentiated RPE cells at approximately 57 kDa (Fig 1I), indicating differential glycosylation of a subset of glycoproteins in myofibroblasatic RPE. Probing sialylation with *Maakia amurensis lectin* (MAL2, data not shown), which more specifically binds α2,3-linked sialic acid, and *sambocus nigra agglutinin* (SNA), which specifically demonstrates the presence of α2,6 linked sialic acids, resulted in binding to numerous, although not identical, bands (Fig 1J). These findings indicate that overall sialylation is not modified during EMT of RPE cells.

Taken together these results suggest that in transdifferentiated, myofibroblastic RPE cells the global N-glycan profile shifts from high mannose-type glycans towards complex-type branched tri- and tetraantennary N-glycans and complex-type biantennary glycans with poly-N-acetyllactosamine chain extensions and terminal N-acetyllactosamine residues. Furthermore, there is evidence for an increased expression of non-sialylated TF-antigen, whereas native and myofibroblastic RPE cells qualitatively but not quantitatively differ in terms of sialylation of glycans.

### Gal-3 binds to the RPE cell surface via β1,6-N-acetylglucosamine(GlcNAc)-branched tri- and tetrantennary complex-type N-glycans but not complex-type O-glycans

**Fig 2:**
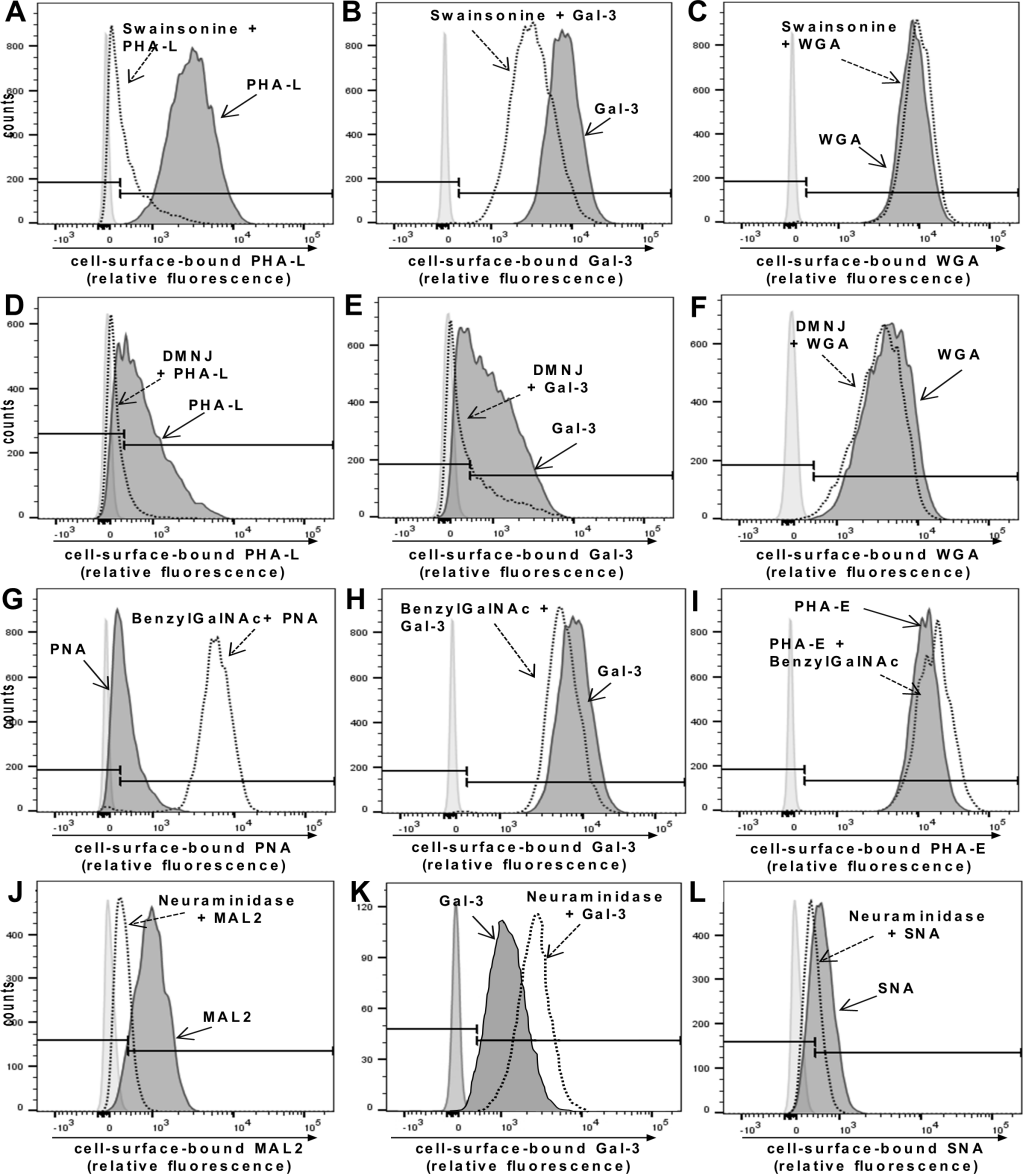
Complex-type N-glycans but not O-glycans are required for Gal-3 binding to the RPE cell surface. (A-C) Cultured human RPE cells were treated with swainsonine to block N-glycan elongation followed by incubation with Gal-3 or plant lectins for 30 minutes at 4°C as described in the Materials and Methods Section. (A) Effectiveness of swainsonine treatment is shown by decreased staining with the plant lectin PHA-L (*dotted line*) compared with control treated cells (*thick line*). (B) Reduction of complex-type N-glycans on RPE cells results in decreased Gal-3 binding (*dotted line*). (C) Binding of the control lectin WGA is not modified. (D-F) Treatment of cultured RPE cells with deoxymannojirimycin (DMNJ), another inhibitor of N-glycan branching, reduces binding of the plant lectin PHA-L (*dotted line*) (D) and Gal-3 (*dotted line*) (E) compared with control treated cells (*thick line*) whereas binding of the control lectin WGA (*dotted line*) is not affected (F). (G-I) RPE cells were treated with BenzylGalNAc, which inhibits elongation of O-glycans and can compete with sialyltransferases resulting in decreased O-glycan sialylation, allowing increased branching of O-glycans. Decreased O-glycan sialylation is shown by increased staining with the plant lectin PNA (*dotted line*) compared with untreated controls (*thick line*) (G). (H) Increased accessibility of branched O-glycans does not alter binding of Gal-3 to RPE cells (*dotted line*), showing that branched O-glycans are not required for Gal-3 binding. (I) Binding of PHA-E as a control lectin is not altered by benzylGalNAc treatment. (J-L) Treatment of RPE cells with neuraminidase increases Gal-3 binding to the surface of cultured human RPE cells. Cultured, myofibroblastic RPE cells were incubated with or without Vibrio Cholera neuraminidase for 30 minutes at 37°C. (J) Binding of MAL-2 (specific for α2,3 sialic acid residues) (*thick line*) and (L) SNA (specific for α2,6 sialic acid residues) (*thick line*) confirms the presence of sialic acid residues on glycans on the RPE cell surface. Both, MAL-2 and SNA binding, is reduced after treatment with neuraminidase (*dotted line*). Binding of Gal-3 (K) to cultured human RPE is increased by removal of sialic acids (*dotted line*). Results are representative for three independent experiments.

Soluble galectin-3 (Gal-3) was previously shown to inhibit PVR-associated cellular events by binding to yet unknown saccharide ligands on myofibroblastic RPE cells [16]. Gal-3 preferentially binds to galactose-terminated saccharide ligands, particularly to lactosamine sequences that can be found on *N*- or *O*-linked glycans on cell surface glycoproteins [15,40,41]. Since our findings indicated that expression of β1,6-branched complex-type *N*-glycans together with unsubstituted TF-antigen is increased upon EMT of RPE cells, we were interested to find out whether these glycomic changes may account for Gal-3 binding. For this purpose we treated myofibroblastic cultured human RPE cells with inhibitors of glycosylation and examined the effects of the inhibitors on binding of Gal-3 to the surface of the cells (Fig 2). To validate the efficacy of the biosynthetic inhibitors we used plant lectin binding as control. Treating cells with swainsonine, an inhibitor of α-mannosidase II, prevents trimming and processing of high-mannose N-glycans, and thus, blocks the formation of complex-type N-glycans by inducing formation of hybrid structures instead [23,42]. The effects of swainsonine were monitored by reduced binding of PHA-L, which recognizes tri- and tetraantennary complex-type *N*-glycans [43]. As shown in Fig 2A, PHA-L bound to the surface of cultured RPE cells. Treatment of myofibroblastic RPE cells with swainsonine markedly reduced PHA-L staining, confirming the effectiveness of swainsonine treatment in reducing lactosamine addition to *N*-glycans, whereas the binding of the control lectin, wheat germ agglutinin (WGA), a plant lectin which recognizes accessible oligomers of N-acetylglucosamine, was barely influenced (Fig 2C). Importantly, Gal-3 binding to cultured RPE cells was reduced in cells treated with swainsonine, indicating that Gal-3 receptors contain β1,6-N-acetylglucosamine(GlcNAc)-branched complex-type N-glycans (Fig 2B). Treatment of myofibroblastic cultured human RPE cells with deoxymannojirimycin (DMNJ), another inhibitor of N-glycan branching, confirmed the findings from swainsonine treatment. DMNJ is an inhibitor of α-mannosidase I, which trims high-mannose structures on *N*-glycans to allow subsequent addition of *N*-acetylglucosamine residues and elongation of lactosamine sequences [44]. Treatment with DMNJ reduced binding of Gal-3 although not as pronounced as found for swainsonine treatment (Fig 2E) and also reduced binding of PHA-L (Fig 2D), whereas binding of WGA was rather enhanced (Fig 2F).

To modify *O*-glycan elongation, we used benzyl-2-acetamido-deoxy-α-D-galactopyranoside (benzylGalNAc) that can modify *O*-glycan elongation in two different ways [45,46]: In cells with low amounts of sialylated *O*-glycans, benzylGalNAc blocks elongation of *O*-glycans beyond the initial lactsoamine residue, an effect that can be detected by reduced reactivity with PNA. In cells with high levels of sialylated *O*-glycans, benzylGalNAc inhibits *O*-glycan sialylation, resulting in increased exposure of non-sialylated, branched *O*-glycans and therefore increased PNA reactivity [47].

As shown in Fig 2G, treatment of myofibroblastic RPE cells with benzylGalNAc increased PNA binding, indicating that the inhibitor treatment favoured the synthesis of nonsialylated *O*-glycans on these cells, whereas benzylGalNAc did not alter binding of Gal-3 (Fig 2H), suggesting that Gal-3 binding to RPE cells is rather mediated by β1,6-N-acetylglucosamine(GlcNAc)-branched tri- or tetraantennary complex-type N-glycans than O-glycans.

In order to probe for an influence of sialylation on Gal-3 binding we next treated cells with *Vibrio cholerae* neuraminidase, an enzyme that cleaves both, α2,3- and α2,6-sialic acid residues, from glycoproteins. The sialic-acid specific plant lectins MAL2 (more specific for α2,3-linked sialic acid) and SNA (specific for α2,6-linked sialic acid) confirmed the presence of sialylated glycans on the surface of dedifferentiated RPE cells. Binding of both lectins was reduced after neuraminidase treatment, corroborating an efficient removal of sialic acid residues (Fig 2J and 2L). Conversely, removal of sialic acids enhanced binding of Gal-3 (Fig 2K). Taken together, these results suggest that inhibition of N-glycan branching alleviates Gal-3 binding to the surface of myofibroblastic RPE cells, whereas reduction of cell surface sialic acid content increases Gal-3 binding. A quantitation of the changes in lectin-surface binding as determined by flow cytometry is provided in S1 Table.

### Inhibition of RPE attachment and spreading by recombinant Gal-3 requires β1,6-(GlcNAc) branched tri- and tetraantennary complex-type *N*-glycans

**Fig 3:**
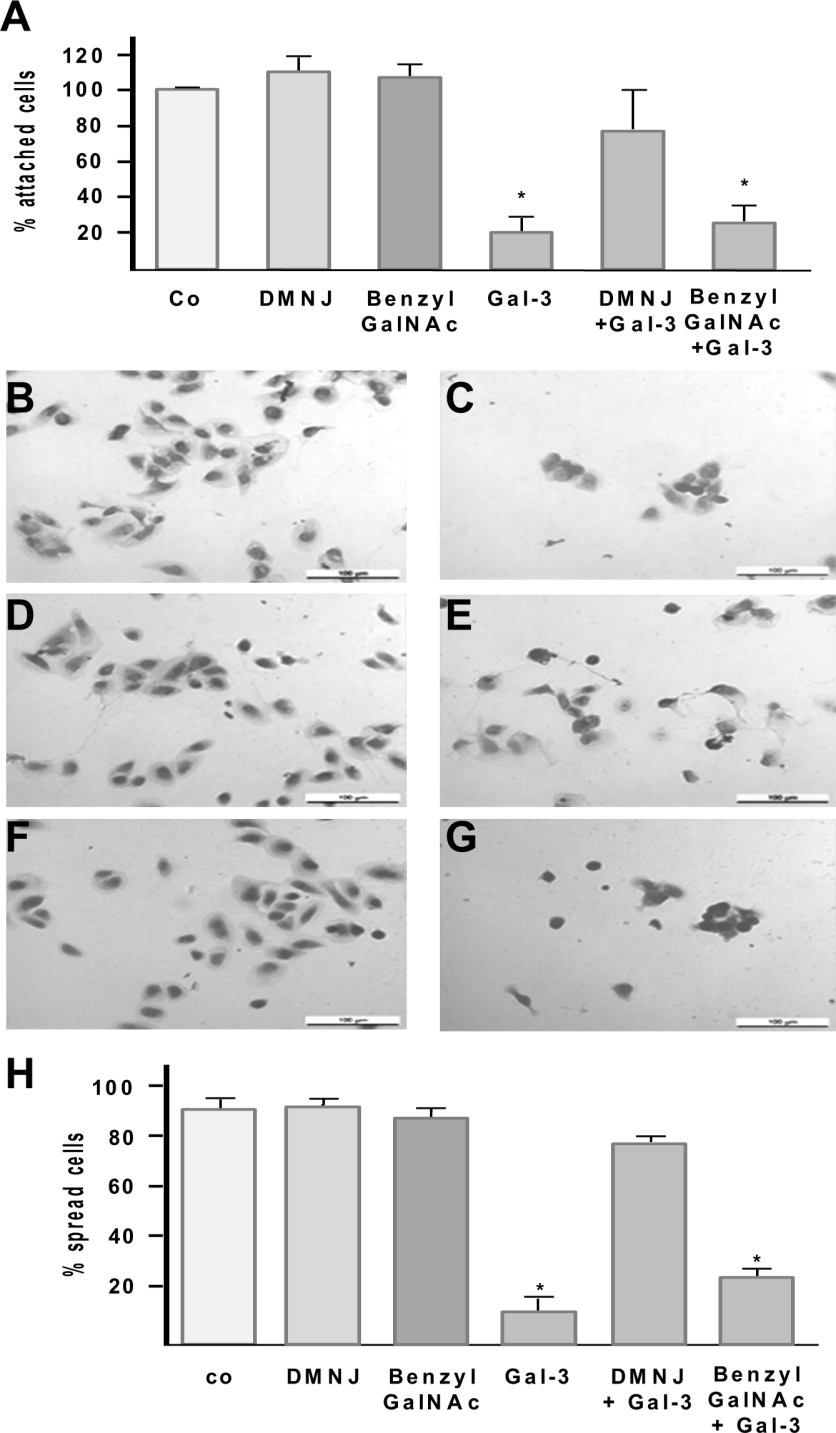
Effect of glycan synthesis inhibitors on Gal-3 mediated inhibition of RPE cell attachment and spreading. Cultured, myofibroblastic human RPE cells were pretreated with or without the *N*-glycan synthesis inhibitor DMNJ or the *O*-glycan synthesis inhibitor benzylGalNAc in the medium for 72 hours. Suspended RPE cells were then incubated with Gal-3 and allowed to attach on fibronectin as described in the Materials and Methods Section (A). The N-glycan synthesis inhibitor DMNJ partially reverses the Gal-3 mediated effect, whereas the O-glycan synthesis inhibitor did not influence the inhibition of RPE attachment by Gal-3. Values indicate means ±SD of four experiments performed in triplicate and are expressed as percentage of controls without galectin-3 in the medium. Co, untreated control. (B-H) For spreading assays suspended RPE cells were pretreated with glycan synthesis inhibitors as described above and then incubated for 35 min with 125 μg/mL galectin-3 (Gal-3) allowed to spread on fibronectin as described in the Materials and Methods Section. Cells were then fixed, stained with giemsa and observed by light microscopy (magnification 20×). Representative light microscopic fields are shown. Exposure of RPE cells to Gal-3 reduces RPE spreading on fibronectin (C), whereas untreated controls (B) and cells pretreated with DMNJ (D) or BenzylGalNAc (F) alone establish broad cytoplasmic halos. Pretreatment with DMNJ partially abolishes the Gal-3 mediated effect (E), whereas benzylGalNAc failed to reduce the Gal-3 mediated inhibition of RPE cell spreading (G). (H) Quantification of cell spreading shown in A-F. Results were obtained from evaluation of five separate fields by examining at least 100 cells per field. Spreading cells were defined as cells with cytoplasmic protrusions and perinuclear halo formation, and non-spreading cells as rounded cells with small extent of cytoplasmic spreading. Values indicate means±SD of three experiments performed in duplicate and are expressed as percentage of total cells present on the respective microscopic field. Statistical analysis was performed using Mann–Whitney U test and a p-value <0.05 was considered as statistically significant (*).

In order to determine whether inhibition of RPE attachment and spreading by Gal-3 requires *N*- or *O*-glycan structures as determined above we treated cultured RPE cells with a myofibroblastic phenotype with either DMNJ or benzylGalNAc, respectively, and assessed whether the glycan synthesis inhibitors modify the capability of Gal-3 to reduce RPE attachment on fibronectin (Fn) (Fig 3A). Treatment of suspended cultured RPE cells with 125 μg/mL recombinant Gal-3 reduced the number of attached RPE cells to 18.7% (±8.1 SD) when compared to untreated controls. Pre-incubation with DMNJ or benzylGalNAc alone for 72 hours had no impact on the attachment rate of the cells. However, while pretreatment with benzylGalNAc did not counteract the Gal-3-mediated reduction of RPE attachment, the inhibitory effect of Gal-3 was significantly reduced when synthesis of complex-type N-glycans had been inhibited by DMNJ (Fig 3A).

Next we tested the influence of DMNJ or benzylGalNAc treatment on Gal-3-mediated inhibition of cell spreading. When compared to untreated controls the capability of myofibroblastic RPE cells to spread on fibronectin was not altered after cells had been pretreated with DMNJ or benzylGalNAc for 72 hours alone (Fig 3B, 3D and 3F). Whereas treatment with benzylGalNAc had no significant effect on the Gal-3 mediated impairment of RPE cell spreading (Fig 3G), treatment of RPE cells with DMNJ causing reduction of complex N-glycans (Fig 3E), partially reversed the inhibitory effect of recombinant Gal-3 on RPE cell spreading with 79.7% (±6.2 SD) of cells establishing a cytoplasmic halo. Thus, polylactosamine sequences on O-glycans are not required for Gal-3 induced inhibition of spreading in these cells. In agreement with findings from flow cytometry and RPE attachment assays these results indicated that β1,6-(GlcNAc)-branched tri- and tetraantennary complex-type *N*-glycans on cell-surface glycoproteins are major glycoligands for Gal-3 on myofibroblastic RPE cells and that Gal-3 binding to these glycoconjugates is required for the Gal-3 mediated functional effects.

### Knockdown of N-acetylgucosaminyltransferase V (Mgat5) expression reduces the ability of Gal-3 to bind to the surface of myofibroblastic RPE cells

**Fig 4:**
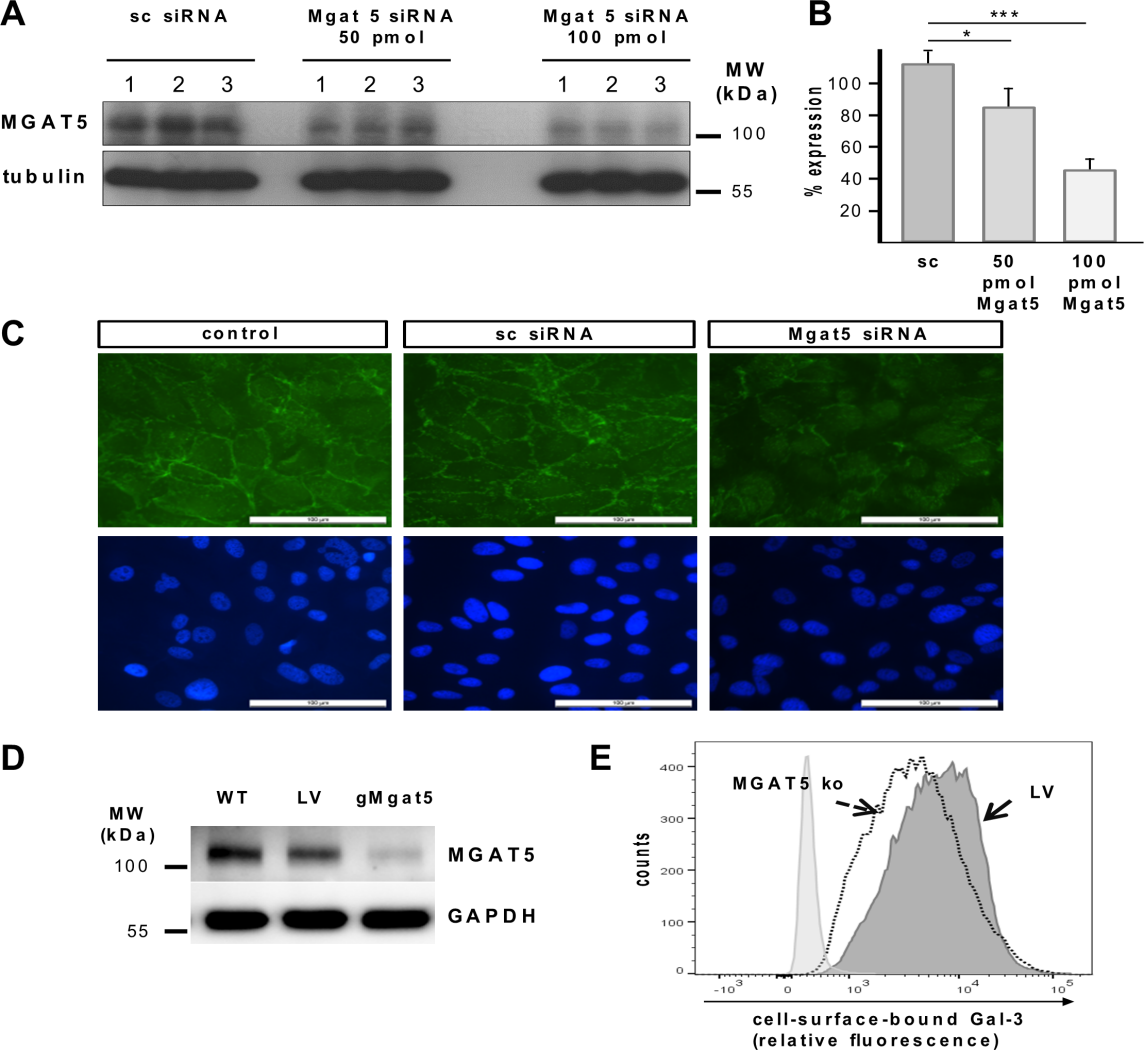
Knockdown of Mgat5 expression in cultured human RPE cells attenuates binding of Gal-3 to RPE cells. (A) Western blot analysis of Mgat5 expression in RPE cells transfected with siRNA containing the same nucleotides as Mgat5 siRNA in random order (sc siRNA), and the same cell line transfected with 50 pmol and 100 pmol of double stranded siRNA complementary to Mgat5 (Mgat5 siRNA), respectively. Lysates containing approximately equal amounts of protein were separated by SDS-PAGE and blotted for immunochemical detection of Mgat5 content. Experiments were repeated at least three times. MW; molecular weight. (B) Quantification of Mgat5 gene silencing. Values are normalized to expression of tubulin. (C) Fluorescence micrographs of Gal-3 binding to the RPE cell surface. Ninety-six hours after transfection cells were treated with 60 μg/mL biotinylated Gal-3. Cells were then fixed and stained with a fluorescent streptavidin conjugate. Nuclei were counterstained with DAPI. Localization of Gal-3 binding was visualized by fluorescence microscopy at a 40 fold magnification. Scale bars represent 100 μm. Untreated cells exposed to streptavidin conjugate alone served as negative controls and exhibited no fluorescence signal (data not shown). (D-E) The target sequence derived from the genomic sequence of Mgat5 was inserted into a CRISPR-Cas9 nuclease expressing lentiviral vector and ARPE19 cells were transfected using Lipfectamine 2000 Plus reagent. (D) Western blot analysis of Mgat5 expression in ARPE19 cells transduced with guide RNA leading to specific knockdown of the Mgat5 (gMgat5), or cells tranduced with a CRISPR-Cas9 lentiviral vector encoding for an none-coding filler RNA (LV), or wild-type ARPE-19 cells. Lysates containing approximately equal amounts of protein were separated by SDS-PAGE and blotted for immunochemical detection of Mgat5 content. (E) Flow cytometric analysis of Gal-3 binding in Mgat5-knockout cells. CRISPR-Cas9-mediated Mgat5 knockdown of cultured RPE cells reduces cells surface binding of Gal-3, when compared to cells transfected with a non-coding control vector alone (LV). Histograms represent the number of counted cells versus relative fluorescence intensity. Transduction experiments have been repeated three times.

β1,6-(GlcNAc)-branched tri- and tetraantennary complex-type *N*-glycans are created in the Golgi by the enzyme N-acetylgucosaminyltransferase V (Mgat5). Mgat5 promotes the formation of N-glycan intermediates, the β1,6-branched *N*-glycans that are then elongated with N-acetyllactosamines to create the high affinity ligands for Gal-3 [48]. To evaluate whether Gal-3 binding is related to Mgat5 expression and to further ascertain the relevance of Mgat5-generated glycans for Gal-3 binding to RPE cells, the expression of Mgat5 was silenced by transient transfection of cultured myofibroblastic RPE cells with siRNA constructs directed against Mgat5. Reduced expression was analyzed by western blot and normalized to the expression of tubulin as a housekeeping gene. Transfection with 50 pmol or 100 pmol Mgat5 siRNA resulted in remaining Mgat5 protein levels of 76% and 41%, respectively, as compared to cells transfected with non-target specific (scrambled) siRNA (Fig 4A and 4B). Consistent with the decreased Mgat5 protein expression levels, cells treated with 100 pmol Mgat5 siRNA showed a reduction of Gal-3 binding as detected by fluorescence staining (Fig 4C). To further substantiate these findings, we generated RPE cells with stable Mgat5-knockdown by CRISPR-Cas9genome editing. For these experiments we used an immortalized human RPE cell line (ARPE19), because primary human RPE cells can only be maintained for a few passages in culture. Fig 4D depicts Mgat5 protein levels for wild-type ARPE-19 cells (wt), ARPE-19 cells transduced with the lentiviral vector expressing a none-coding filler guide RNA (LV), and a gMgat5-CRISPR/Cas9 knockout clone (gMgat5) sixteen days after transduction. CRISPR-Cas9-mediated Mgat5 knockout significantly reduced Mgat5 protein expression levels when compared to wild-type or control transfected cells. We then examined the effect of Mgat5-knock-down on the binding of Gal-3 to the transduced versus non transduced cells by flow cytometry. The binding of Gal-3 (Fig 4E) decreased in Mgat-5 knockdown cells, which confirmed findings from siRNA experiments suggesting that Mgat5-modified β1,6-(GlcNAc)-branched tri- and tetraantennary complex-type *N*-glycans are required for Gal-3 binding to myofibroblastic RPE.

### Expression of Mgat5 increases upon EMT in human RPE cells conferring increased binding of Gal-3

**Fig 5:**
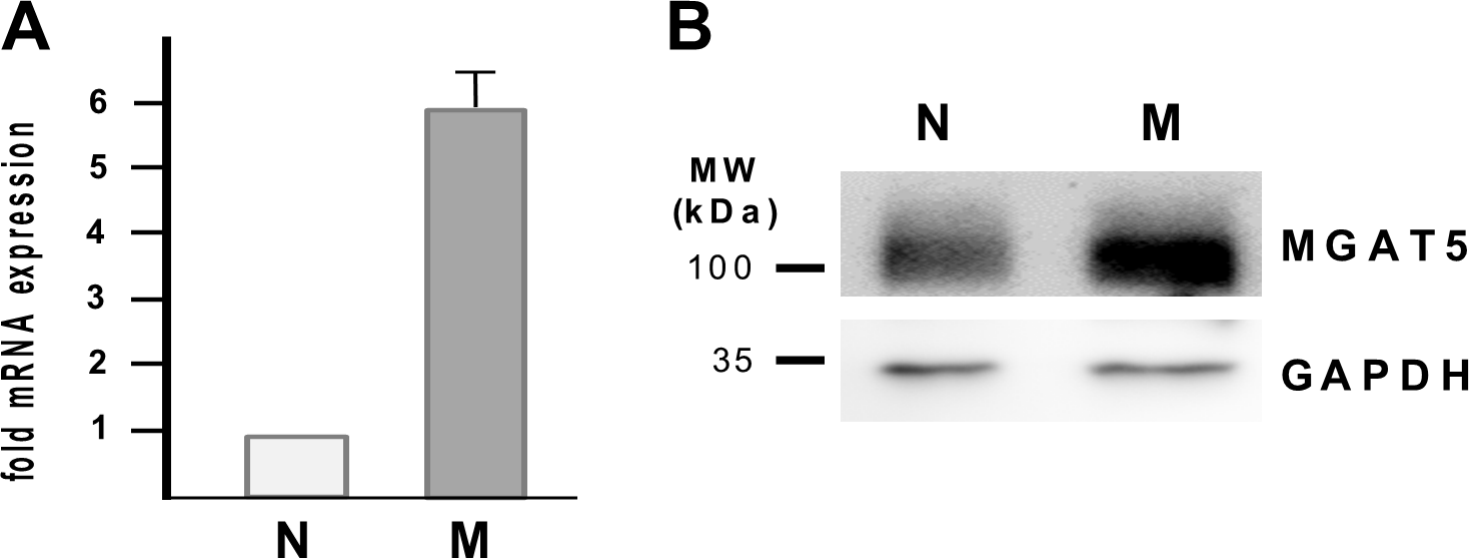
Upregulation of Mgat5 and β1,6GlcNAc-branched N-glycosylation in myofibroblastic RPE. (A) Total cellular RNA from native (N) and cultured, myofibroblastic human RPE cells (M) was analysed for β1,6-*N*-acetylglucosaminyltransferase (Mgat5) expression levels by quantitative real-time RT-PCR as described in the Materials and Methods Section. Data are presented as relative Mgat5 mRNA expression levels as compared with native RPE cells and represent the mean ± SD of at least 3 experiments. (B) Equal amounts of whole cellular protein lysates (50 μg) derived from native (N) and cultured, myofibroblastic (M) human RPE cells of passage 3 were separated by 10% SDS PAGE and Mgat5 levels detected by Western blot. Equal loading was confirmed by GAPDH. Experiments were repeated at least three times

**Fig 6:**
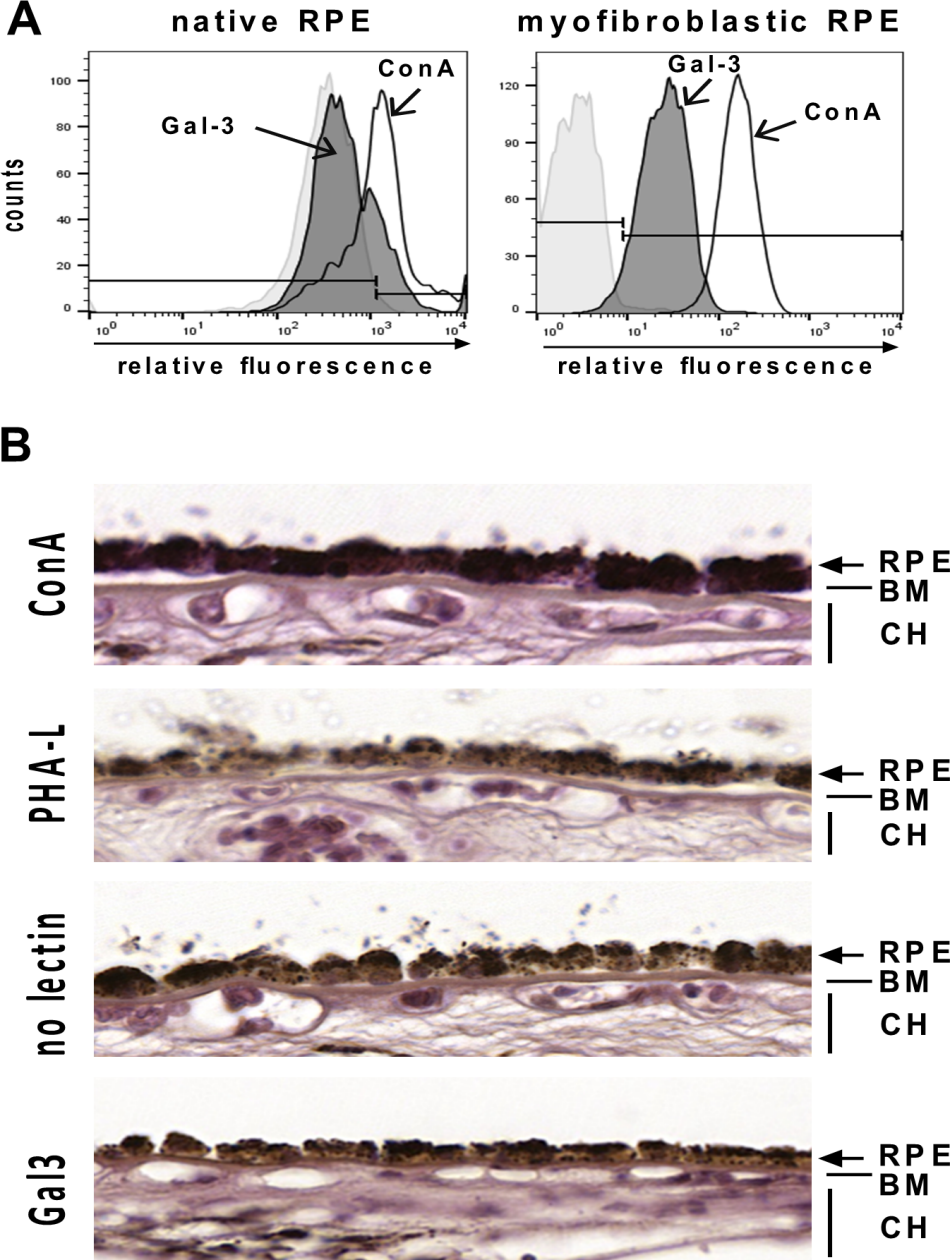
Native RPE cells exhibit little binding of Gal-3. (A) Flow cytometric analysis of Gal-3 binding to native and myofibroblastic RPE cells. Binding of Gal-3 (*gray*) to native RPE cells was only slightly above background, whereas Gal-3 binding to myofibroblastic RPE cells was evident. ConA binding was markedly above background in both cell populations. Histograms represent the number of cells versus relative fluorescence intensity. Experiments have been repeated two times. (B) Lectin histochemistry of human RPE cells in situ. Human cadaver eyes were incubated with biotinylated lectins as indicated on the left and binding was visualized by incubation with streptavidin-coupled peroxidase and Vector VIP substrate™ (purple color). In control sections with the VIP substrate alone the RPE can be easily discerned by the characteristic brownish pigment (third panel from the top). RPE cells and extracellular matrix reacted strongly with ConA *(top panel*), whereas PHA-L (*second panel* from the top) did not recognize native RPE cells and exhibited a staining pattern comparable to that of substrate alone. Biotinylated Gal-3 (purple color, *fourth panel*) did not bind to native RPE in situ and staining patterns resembled closely the situation in untreated negative control eyes. RPE, retinal pigment epithelium; BM, Bruch´s membrane; CH, choriocapillaris.

Having shown that EMT of RPE cells is characterized by an abundance of tri- and tetraantennary complex-type N-glycans on the surface of RPE cells and that functional effects and binding of soluble Gal-3 to the RPE cell surface require these Mgat5 modified glycans, we hypothesized that expression levels of Mgat5 may change upon EMT of RPE cells *in vitro* and that Gal-3 for this reason may with relative selectivity bind to myofibroblastic RPE cells. As evidenced by quantitative real-time RT-PCR myofibroblastic RPE cells exhibited an approximately 5.85 fold (±0.78 SE) upregulation of Mgat5 mRNA as compared to native RPE cells (Fig 5A). This increase in Mgat5 mRNA translates into increased Mgat5 protein levels with 2.5 fold higher Mgat5 expression levels in myofibroblastic, cultured RPE cells compared to native, differentiated RPE cells (Fig 5B), which may at least partially account for the increased expression of β1,6-(GlcNAc)-branched tri- and tetraantennary complex-type *N*-glycans upon EMT. To confirm the hypothesis that native RPE cells thus have less affinity for Gal-3 flow cytometry was performed. In native RPE cells we found strong background fluorescence due to their intense pigmentation. While ConA binding (positive control) pronouncedly increased fluorescence intensities on native RPE, incubation with Gal-3 did not alter the level of background fluorescence (Fig 6A, *left panel*) suggesting a lack of Gal-3 binding. In contrast, in myofibroblastic RPE cells (Fig 6A, *right panel*) pronounced Gal-3 binding was evident. In an attempt to strengthen these results we next assessed the potential of PHA-L, ConA and Gal-3 to bind to native RPE cells still residing in the eye cup by histochemistry. Posterior poles from human cadaver eyes were treated with biotinylated Gal-3, PHA-L or ConA and sections of the RPE/choroid sclera specimen were then evaluated by light microscopy (Fig 6B). Whereas RPE cells *in situ* exhibited a pronounced reactivity with ConA, the staining pattern of PHA-L strongly resembled that of streptavidin alone (negative control), which is in agreement with the observations from lectin blots and flow cytometric analyses. Sections from eyes that had been incubated with biotinylated Gal-3 prior to fixation showed purple staining in distinct RPE cells, whereas other RPE cells remained unstained very similar to the binding patterns of PHA-L and the negative control. These observations confirm the notion that native RPE cells to a lesser extent than cultured, myofibroblastic RPE bind PHA-L because they harbour only little β1,6-N-glycosylation. The comparable binding pattern of Gal-3 in some cells indicates that native RPE cells also have little affinity for Gal-3, when the cell membranes are intact. Positive staining of distinct cells may originate from the interaction of Gal-3 with non-glycosylated intracellular binding partners or cytokeratins in cells with cell membrane damage.

## Discussion

We demonstrate here for the first time that human RPE cells upon epithelial-to-mesenchymal transition (EMT) acquire a unique glycan expression profile that is distinct from healthy, native RPE cells and provide evidence that myofibroblastic RPE cells express an abundance of non-sialylated Thomsen Friedenreich (TF) antigen as well as complex-type β1,6-branched tri- and tetraantennary *N*-glycans (β1,6(GlcNAc)-branched complex-type N-glycans), glycan chain extension with poly-*N*-acetyllactosamine, and terminal N-acetyllactosamines when compared to native RPE cells.

These findings indicate that the glycomic profile of human RPE cells undergoes a profound reorganization upon RPE EMT in vitro. Certainly, binding of the plant lectin probes to immobilized glycoproteins in lectin blots does not favor conformation-dependent protein-protein interactions and given the diversity in the cellular glycome resulting from glycan branching, chain extensions and variations with polylactosamine, fucose, sulfate and sialic acid or methylation and acetylation we in this approach monitored only a small aspect of the glycan structures. Nevertheless, we were able to detect profound changes of the RPE glycan expression patterns upon RPE dedifferentiation in vitro and our findings fit well into the overall theme of glycomic changes associated with EMT. We found evidence for an abundance of complex-type β1,6-branched complex-type *N*-glycans, poly-N-acetyllactosamine elongations [41], as well as PNA-reactive bands which may reflect an increase of non-sialylated Thomsen Friedenreich (TF) antigen in myofibroblastic RPE cells [39]. These glycomic changes can frequently be observed in premalignant and malignant epithelia [49]. With respect to RPE cells however, this is a completely new finding. The TF antigen is the core 1 structure of mucin-type O-glycans, but in its simplest, non-sialylated, non-extended form the TF-antigen acts as an oncofetal antigen and is supposed to play a role in cancer cell adhesion [50]. Accumulation of β1,6-(GlcNAc)-branched *N*-glycans containing poly-*N*-acetyllactosamine is highly correlated with carcinoma progression [51–54], although this does not apply to all types of tumors. In hepatocellular carcinoma and non-small cell lung cancers, for example, low expression levels of β1,6-branched *N*-glycans and Mgat5 are associated with poor prognosis, suggesting tissue-specific effects [55]. This far, only few studies have focused on the role of differential glycosylation in nonmalignant tissues. For example, β1,4-galactosyltransferase-1, which synthesizes the type 2 chain (Galβ1,4GlcNAc) on N-glycans and the core 2 branch in O-glycans, has been shown to participate in skin wound healing. In the eye, Saravanan et al. reported an upregulation of T-synthase, which generates Thomsen-Friedenreich (TF)-antigen, another known ligand for Gal-3 [24,49], together with a downregulation of several sialyltransferases in murine healing corneas. In agreement with our observations, they also observed an increased β1,6-N-glycan branching. However, as opposed to our findings, this appeared to be associated with downregulation of N-acetylglucosaminyltransferase III (Mgat3), which introduces bisecting β1,4GlcNAc and thereby suppresses β1,6GlcNAc branching by Mgat5, while expression of Mgat5 was not upregulated. This is in clear contrast to our findings as we found that the increase of β1,6-branched *N*-glycans in myobroblastic RPE cells is associated with an upregulation of Mgat5. Because it is assumed that Mgat3 dominantly competes with Mgat5 for the modification of the same protein the authors proposed that downregulation of Mgat3 may result in increased β1,6-branching by Mgat5 and affinity for Gal-3 [36]. Thus, tissue-specific regulatory mechanisms for glycan expression may also exist in non-malignant tissue. Whether the differentially expressed glycan epitops found to be abundant on myofibroblastic RPE cells are instrumental for the abnormal behavior of RPE following EMT and maintenance of the myofibroblastic phenotype is subject of ongoing studies. To this date treatment of myofibroblastic RPE cells with inhibitors of glycosylation according to the protocol used in the present study neither altered the phenotype nor RPE attachment and spreading on fibronectin. In fact, glycosylation-dependent functional effects appeared to require the action of a carbohydrate-binding protein, namely Gal-3.

Several lines of evidence suggest that Gal-3 is the missing link between the presence of Mgat5-modified complex-type *N*-glycans on distinct cell surface glycoprotein receptors and the altered behavior of the cells in malignant and premalignant tissues. Soluble Galectin-3 (Gal-3) inhibits attachment and spreading of cultured human RPE cells in a carbohydrate-dependent manner and is upregulated in myofibroblastic RPE [16]. Whereas some protein ligands on RPE cells recognized by Gal-3 have been identified [28], Gal-3 recognition of specific glycans on RPE cells remained unknown. To find out whether the glycomic shift accounts for Gal-3 binding to myofibroblastic RPE and to identify the binding determinants of Gal-3 on myofibroblastic RPE cells we chose to employ flow cytometry and evaluated Gal-3 binding towards the RPE cells in a concentration range, which functionally elicits inhibition of RPE attachment and spreading [16]. As evidenced by the positive binding of the plant lectins MAL-2 and SNA, which recognize α2,3 and α2,6 sialic acid residues, respectively, flow cytometry confirmed lectin blot analysis showing that myofibroblastic RPE cells carry considerable amounts of sialylated glycans on the cell surface. This is further substantiated by the enhanced binding of PNA after incubation with benzylGalNAc, which interferes with O-glycan elongation but can also compete with sialyltransferases and thus lead to an increase of non-sialylated O-glycans accessible for the core 1 O-glycan binding PNA [47]. Recent studies have demonstrated that sialylation of glycans distinctively modulates the recognition of cell surface glycans and biological responses triggered by galectins [25]. Fittingly, enzymatic desialylation of the cells appreciably enhanced Gal-3 binding, while binding MAA and SNA was markedly reduced, suggesting that sialylation is another key regulator of RPE sensitivity to Gal-3. This finding is consistent with previous studies in colon adenocarcinoma cells demonstrating that although Gal-3 preferentially binds to unsialylated glycans, it can tolerate terminal α2,3 and α2,6 sialic acid residues and bind to the cells [56]. This is in clear contrast to other galectin-subtypes, namely Galectins-1 and -2 (Gal-1, Gal-2), which do not bind when glycans are terminally sialylated [25]. The finding that the increase in non-sialylated complex O-glycans after benzylGalNac treatment did not enhance Gal-3 binding, further substantiates the notion that in dedifferentiated RPE O-glycans play a minor role for the affinity of Gal-3 to the RPE, although several reports provide evidence of Gal-3 binding to O-linked glycans such as those present in CD43 and CD45 or MUC1 [49,57]. The most striking finding from these experiments was that treating cells with swainsonine and deoxymannonjirimycin (DMNJ), which inhibit enzymes necessary for trimming and processing of high mannose-type N-glycans and thus block the formation of β1,6-*N*-acetylglucosamine(β1,6GlcNAc)-branched complex-type N-glycans, reduce binding of Gal-3 to the RPE cell surface and the capacity of Gal-3 to inhibit attachment and spreading of RPE cells, suggesting that β1,6GlcNAc-branched complex-type N-glycans are functionally relevant binding determinants for Gal-3. Gal-1 is another galectin that has been shown to inhibit attachment and spreading of RPE cells [32]. In an earlier study we found that pretreatment of RPE cells with Gal-1 blocks Gal-3 binding to the RPE cell surface and that simultaneous treatment with Gal-1 and Gal-3 has no additive inhibitory functional effect [16]. In conjunction with the observation that Gal-1 binding to the RPE surface is reduced upon inhibition of mannosidase I (unpublished data) these findings may suggest that the two lectins share a certain subset of cell surface carbohydrate counterreceptors. Precisely, to what extent Gal-1 and Gal-3 share counterreceptors remains to be determined.

The biosynthesis of β1,6-branched complex-type *N*-glycans critically depends on the activity of N-acetylglucosaminyltransferase V (Mgat5). Mgat5 catalyses the linkage of a N-acetyllactosamine to a core mannose of N-glycan to produce the GlcNAc-β1,6-Man branch, which is then extended to form tri- or tetraantennary oligosaccharide chains [55]. Tumor cells from Mgat5^-^/^-^ mice fail to undergo epithelial-to-mesenchymal transition and are insensitive to multiple cytokines, including epidermal growth factor (EGF), patelet-derived growth factor (PDGF) and transforming growth factor beta (TGF-ß) [41]. In the view of these findings the most important observation from the present study is that Mgat5 is upregulated upon EMT of RPE cells and that it is functionally active as evidenced by the abundance of β1,6-branched tri-and tetrantennary N-glycans in myofibroblastic but not in native RPE. The relevance of Mgat5-modified β1,6-branched glycans for Gal-3 binding to RPE cells is further substantiated by the reduced binding of Gal-3 to cultured RPE with low Mgat5 expression levels.

This is of particular importance since in breast carcinoma cells Gal-3 cross-links tri-and tetraantennary complex-type *N*-glycans on EGF receptor, PDGF receptor and TGFβ receptors [41], and in endothelial cells Gal-3 reacts with complex-type N-glycans on vascular endothelial growth factor receptor 2 [58] thereby delaying their removal by constitutive endocytosis. Binding to the respective glycans on α3β1 integrin [15] was shown to promote lamellipodia formation in corneal epithelial cells. On T-cell receptors Gal-3 binding to complex type N-glycans opposes antigen-dependent clustering and suppresses autoimmune disease [41,51], and in RPE cells we in a previous study observed clustering of the β1,6-branched complex-type *N*-glycans bearing Gal-3 ligands CD147 and α3β1 integrin upon addition of Gal-3 [28]. Of note, most of these glycoprotein receptors have been shown to play a role in the pathogenesis of PVR [2,3,59–68]. This study focused the overall cell surface glycan expression profile responsible for Gal-3 binding, but it will be of high interest to trace the differential β1,6-N-glycosylation down to individual proteins. Clearly, this issue awaits further investigation.

Having defined the functionally most relevant oligosaccharide ligand of Gal-3 in RPE cells together with the abundance of the respective glycans on myofibroblastic but not on native, healthy RPE cells, one major goal of the present study was to evaluate, whether this confers binding selectivity for the myofibroblastic phenotype of RPE cells. Direct comparison of the two RPE phenotypes by FACS analysis confirmed the Gal-3 binding in favour of myofibroblastic RPE when compared to native RPE. This was also evidenced by lectin histochemistry of human cadaver eyes. PHA-L failed to bind to RPE cells residing on Bruch´s membrane, whereas the cells were intensely stained by ConA, which was consistent with the findings from lectin blot analysis. Admittedly, Gal-3 appeared to stain the cytoplasm of individual cells, whereas others remained unstained. However, this differential reactivity of PHA-L and Gal-3 with native RPE cells could arise from the high specificity of PHA-L for tri- and tetraantennary branched complex type-N-glycans, whereas Gal-3 reportedly has ligands inside the cells, such as the nonglycosylated proteins gemin 1 and 4 or β-catenin [69,70], but also to terminal N-acetylgalactosamine residues on cytokeratins [71]. In this respect it is noteworthy that most cadaver eyes obtained for the present study had postmortem times of more than 10 hours. Autolytic processes together with the manipulation when removing the retina may have permeabilized some RPE cells allowing for penetration of biotinylated Gal-3 and binding to the intracellular ligand structures. Thus, despite of these constraints we propose that recombinant Gal-3 binds to the myofibroblastic phenotype of RPE cells with relative selectivity.

To the best of our knowledge this study provides the first evidence that EMT of RPE cells *in vitro* confers glycomic changes and that these changes are associated with an increased responsiveness to Gal-3. In contrast to healthy, native RPE cells the myofibroblastic phenotype of RPE cells expresses an abundance of β1,6-branched complex N-glycans, the high affinity ligands for Gal-3, and interaction of Gal-3 with theses glycans interferes with RPE attachment and spreading. Given the relative selectivity of Gal-3 for the myofibroblastic RPE phenotype together with its capability to inhibit early PVR-associated cellular events it is tempting to speculate that from a therapeutic perspective targeting β1,6(GlcNAc)-branched *N*-glycans by recombinant Gal-3 or other carbohydrate-based drugs may allow to selectively target transdifferentiated cells present in the vitreous and thus provide a novel concept for prophylaxis of PVR. Further investigations on this pathway may ultimately help us to exploit galectins for their therapeutic purposes in future drug discovery for treatment of PVR and other RPE-associated proliferative vitreoretinal disorders.

## Supporting Information

S1 Table Quantitative data on the changes of mean fluorescence intensity cells labelled by biotinylated Gal-3 and plant lectins as shown in Fig 2. % parent, percentage of cells out of the parent gate (percent parent). CV, coefficient of variation. MFI, mean fluorescent index. Results are calculated from three independent experiments. Statistical analysis was performed with Mann–Whitney U test and a p-value <0.05 was considered as statistically significant (*).
(TIF)
