# Supplementary figures and images for: Epithelial-to-Mesenchymal Transition of RPE Cells In Vitro Confers Increased β1,6-N-Glycosylation and Increased Susceptibility to Galectin-3 Binding
Source: PLoS One. 2016 Jan 13;11(1):e0146887. doi: 10.1371/journal.pone.0146887 (PMC4712018; doi:10.1371/journal.pone.0146887)

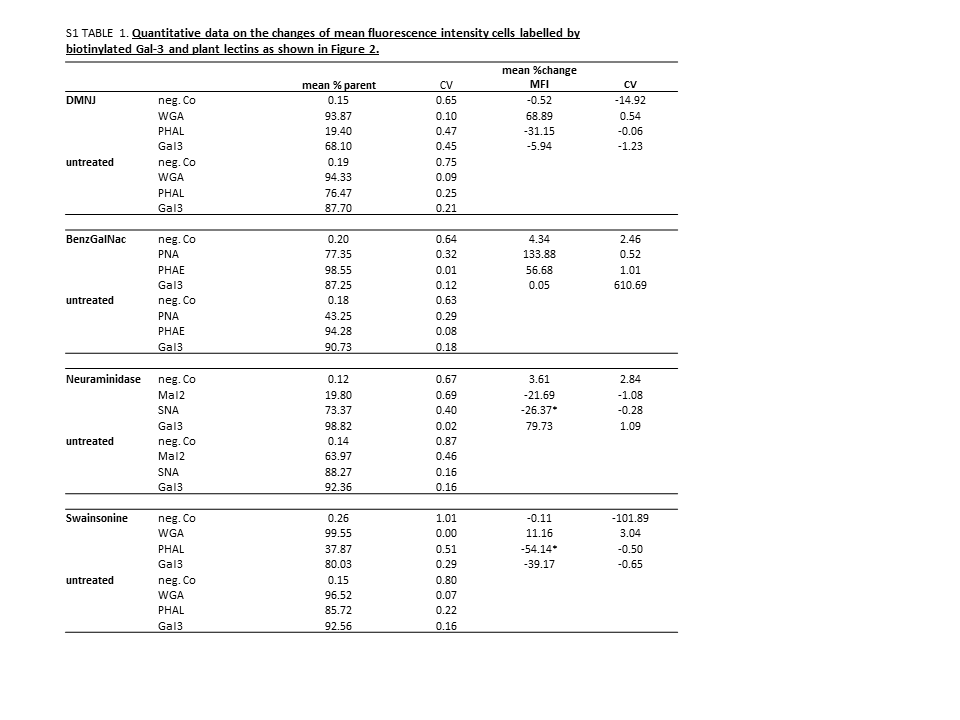

Supplement: S1 Table — % parent, percentage of cells out of the parent gate (percent parent). CV, coefficient of variation. MFI, mean fluorescent index. Results are calculated from three independent experiments. Statistical analysis was performed with Mann–Whitney U test and a p-value <0.05 was considered as statistically significant (*). (TIF) [file pone.0146887.s001.tif]
